# Influence of intermittent iron and folic acid supplementation on cognitive abilities among adolescent girls in northwestern Tanzania

**DOI:** 10.1371/journal.pgph.0002079

**Published:** 2023-10-18

**Authors:** Yasinta Bahati, Elias C. Nyanza, Moses Asori, Rita Mutayoba, Deborah S. K. Thomas

**Affiliations:** 1 Department of Environmental, Occupational, and Department of Environmental, Occupational Health, and GIS, School of Public Health, Catholic University of Health and Allied Sciences, Mwanza, Tanzania; 2 Amref Health Africa and Nutrition International, Regional Health Management Team-Simiyu Region, Simiyu Region, Tanzania; 3 Department of Geography and Earth Sciences, University of North Carolina at Charlotte, Charlotte, North Carolina, United States of America; Indian Institute of Technology Bombay, INDIA

## Abstract

Iron and folic acid (IFA) supplementation to reduce anemia is key for improving substantial lost disability adjusted life years (DALYs) for adolescent girls. This study assessed the impact of weekly IFA supplementation (WIFAS) on cognitive ability among adolescent girls in the Simiyu Region in northernwestern Tanzania. This cross-sectional comparative evaluation study of 770 adolescent girls (396 –WIFAS supplemented; 374 –not supplemented) evaluated the association between WIFAS and cognitive ability through a face-to-face survey and cognitive ability assessment using standardized tests (Span-forward Test, Span-backward Test and Maze Test). Using a modified Poisson regression, we controlled for the geographic setting (urban vs rural), availability of potable water and feeding programs in schools, age and school level of adolescent girls, parental status, main parental economic activities, and the number of teachers. Participants were between the ages of 11 and 19 years, with more than half (57%) between 12–15 years of age. Those with WIFAS had higher cognitive ability (Span-forward scores, *χ*^2^ = 46.34% p <0.001; Span-forward, *χ*^2^ = 46.34% p <0.001; and Global Composite Cognitive Performance (GCCP), *χ*^2^ = 32.52% p<0.001). Among the IFA supplemented adolescent girls, secondary school level had a significantly higher score with respect to Span-backward (aPR = 1.43, 95% CI = 1.06–1.62); Span-forward ability (aPR = 1.26, 95% CI = 1.04–1.53) and Maze Test ability (aPR = 1.12, 95% CI = 1.01–1.25) as compared to their counterpart in primary school level. Individual adolescent girls with WIFAS and living with both parents performed much better on the Span-backward Test (aPR = 1.22, 95% CI = 1.07–1.68) as compared to those living with relatives and/or orphans. The presence of potable water program among the WIFAS schools resulted in a higher Span-backward ability (aPR = 1.34, 95% CI = 1.03–1.89); and GCCP (aPR = 1.27, 95% CI = 1.03–1.75). Adolescent girls from WIFAS schools with feeding program had higher Span-forward (aPR = 1.38, 95% CI = 1.03–1.63) ability as well as a higher Maze Test (aPR = 1.15, 95% CI = 1.07–1.26) score. The present study provides compelling evidence that WIFAS is positively associated with higher cognitive ability among adolescent girls. Nevertheless, IFA interventions are still rare in communities across Tanzania.

## 1. Introduction

Iron and folic acid (IFA) supplementation to reduce anemia is key for improving substantial lost disability adjusted life years (DALYs) for adolescent girls [1–3]. Iron and folic acid (IFA) are critical nutrients for blood synthesis and neurocognitive development [4]. A deficiency of these nutrients leads to anaemia, which in turn causes a reduction in oxygen delivery to the brain and hence poor brain development [5, 6]. Poor brain development has been associated with reduced total brain volume, poor neurogenesis, and altered cortical thickness, contributing to diminished short memory retention, attention, intelligence, and sensory functions [7]. Folic acid, a water-soluble B-complex vitamin, has been linked to increased cognitive ability and reduced memory loss [7–10]. Evidence from both human and animal models indicates individuals with low serum folic acid levels are likely to have higher levels of homocysteine, which in turn results in a risk of memory loss of up to 90% [11, 12]. Increased serum folic acid has been reported to improve cognitive ability by reducing homocysteine levels [13].

Adolescent girls have a high demand of iron and folic acid due to the high turnover of nutrients during this critical developmental time for brain and body development [14]. During adolescence, iron deficiency decreases brain iron stores and impairment of iron-dependent enzymes activities necessary for the synthesis, function, and degradation of neurotransmitters [14]. Together with rapid development, other contributing factors to IFA deficiency include poor nutrition, diseases like malaria, worm infestation/hemoglobinopathies, and/or menstruation [15, 16]. As a result, adolescent girls can benefit substantially from IFA supplementation to alleviate anaemia, as multiple bios (de)generative processes are ongoing [17, 18].

An estimated one-quarter of adolescents in developing countries are anaemic [19]. In Tanzania, the 2015–2016 Tanzania Demographic and Health Survey [20], indicated that about 47% of adolescent girls in Tanzania had anemia [20], highlighting the potential for reduced cognitive ability. Many studies throughout the world have shown that IFA supplementation is significantly associated with improvement in cognitive ability among adolescent girls [21–23]. For instance, a systematic review and meta-analysis of older children and adults conducted found that regardless of baseline iron level, taking iron supplements increased focus and attentiveness. Additionally, anaemic participants had a 2.5-point higher IQ following oral iron supplementation [24].

Studies of the effects of IFA supplementations on cognitive abilities among adolescent girls are scarce in most developing countries, including Tanzania. IFA supplementation programming in Tanzania is focused mainly on pregnant women to improve maternal neonatal outcomes. At antenatal care clinics, pregnant women are provided with IFA as part of standard prenatal care. While this important effort is integrated into antenatal care, there is not the same systematic attention on adolescent girls.

The present study leveraged the work of a pilot project already underway in the Simiyu Region in northwestern Tanzania that provided intermittent IFA supplementation to adolescent girls aged 10–19 years of age. In 2022, we evaluated cognitive ability of adolescent girls in the pilot community and in a comparable community without IFA supplementation to assess outcomes and risk factors.

## 2. Methodology

### 2.1 Study setting, study design, and study population

From March 2017 to December 2019, the Tanzanian Ministry of Health (TMH) in collaboration with Nutrition International (NI) and Amref Health Africa (AHA) piloted providing intermittent Weekly IFA supplementation (WIFAS) for adolescent girls aged 10–19 in Meatu District, in Simiyu Region in northern Tanzania. This aimed at addressing iron deficiency anaemia as a response to the TDHS 2015–2016 report which indicated a high prevalence of iron deficiency anemia among women of reproductive age (15–45 years of age) in the Simiyu Region as compared to the national average of the same age group (53.4% vs 45%).

This cross-sectional comparative evaluation study was conducted in Simiyu Region in northwestern Tanzania in September–October 2019 before the completion of the pilot project in December 2019 to compare the cognitive performance among adolescent girls in areas that were supplemented as compared to non-supplemented areas. The study involved two districts Meatu (an IFA supplementation area) and Itilima (a non-IFA supplementation area) in Simiyu Region. The use of a comparison group of adolescent girls from a non-IFA supplementation area increased the robustness and confidence of our findings by allowing us to document the cognitive ability in a population without a supplementation program. Meatu and Itilima Districts had a total population of 94,982 and 95,203 women of reproductive age, respectively [20]. Both Meatu and Itilima Districts have similar characteristics in terms of women of reproductive age population size (94,982 and 95,203), number of schools, and economic activities in both communities [20].

The study recruited 770 adolescent girls aged 10–19 years (n = 396 in Meatu, Supplemented areas, and n = 374 in Itilima non-supplemented areas). The minimum sample size for a cluster sampling was determined based on Paulson et al [25].

Among 770 study participants, 269 were aged 10–14 years (59.11%, n = 159 supplemented; and 40.89%, n = 110, non-supplemented), and 501 were aged 15–19 years (41.31%, n = 237 supplemented; 52.69%, n = 264 non-supplemented). A two-stage cluster sampling technique was employed in the recruitment of the study participants, using the selection of units based on the Probability Proportional to Size (PPS) for drawing the *Primary Sampling Units* (PSU) (See *S1 Table*). The first stage of recruitment involved the selection of schools based on the number of adolescent girls 10–19 years. The second stage involved a systematic selection of adolescent girls where every 5^th^ adolescent girl on the list at the school level was invited to participate. None of the invited declined to participate or withdrew from participation.

The WIFAS Program involved 270 school health teachers (Trainers of Trainees) from secondary and primary schools in Meatu who were trained to facilitate the rollout of Weekly IFA Supplementation in schools. The trained health teachers were equipped with guidelines and manuals on weekly IFA supplement delivery, linkages with health facilities for IFA supplement refill, and weekly dispensing of a single tablet of IFA supplements (Dried Ferrous Sulphate BP 200mg equivalent to 65gm of Iron and Folic acid Bp 0.25mg) per adolescent girl using a *Direct Observation Therapy* (DOT) approach for non‐pregnant women of reproductive age [26]. The school matron supervised the consumption of the tablets every week. To obtain sociodemographic and socioeconomic information, all participating adolescent girls completed a structured pre-tested questionnaire in form of face-to-face interview.

### 2.2 Cognitive performance tests

Cognitive abilities across four cognitive patterns (attention and short-term memory retention; working memory and thinking and acting quickly; solving novel problems; and encoding short-term memory) were assessed using a Digit Span Test (forward and backward, see *S2 Table*) and a Maze Test adopted from the Wechsler Intelligence Scale as applied in a study in India [27]. The average pass mark was 80% for both tests [27]. In the Digit Span Test, lists of numbers were read forward and backward; participants were then asked to repeat them. Forward digit sequence assesses both attention and short-term memory and backward measures working memory. In the Maze Test, participants were provided with a short story with missing words were asked to identify the correct words among provided alternatives to fill in the missing words in the story within 15 minutes. This test assesses the ability to think and act quickly, solve novel problems, and encode short-term memories. Each correct word was scored as 2 points and 80% was a pass mark for participants. In summary, cognitive ability was measured along three domains (i) Span-backward (ii) Span-forward, and (iii) Maze Test to generate outcome variables. Participants were classified as follows: 1) normal outcome if they performed ≥ 90th percentile level on all the test items in that specific test; or 2) impaired if they performed < 90th percentile on the test items in the specific test. Adolescent girls were also classified in term of a composite cognitive score—*Global Composite Cognitive Performance* (GCCP)—computed by the aggregation of the three domains [27], where individual adolescent girls who displayed a normal outcome on all three tests (i.e., Span-backward, Span-forward, and Maze Test) were classified as having normal performance and, those who failed on at least one test were classified as displaying cognitive impairment.

### 2.3 Quality control

Before data collection, the questionnaires were pretested in a different population in Busega District in the same region. The pre-testing aimed at checking 1) the flow of questions and consistency; 2) the understandability of questions; and 3) the timing for completion of the cognitive assessment. To increase the triangulation of the assessment, two research assistants were trained and completed the cognitive assessment for all the recruited adolescents in both districts. All the study tools were translated into the Swahili language–the common language of most Tanzanians. This language was used to reduce language bias and increase comprehension of the assessment and communication during the assessment.

### 2.4 Statistical analysis

Data were cleaned and analyzed using Stata version 15.0 Stata Corp LP [28]. A preliminary exploration of the data was done to check for missing values, duplicates, and unusual observations before analysis. The exposure variables included: age, education level, economic activities of the parents, parent status, residency, availability of water at school, availability of food at school, and number of teachers at school stratified by IFA. The parental status variable was combined (living with relatives and orphans) to increase the power of the groups. The age of participants and the number of teachers at school were categorized for better interpretation. The independent variable was IFA supplementation and response variables included Span Forward and Backward Test scores, and Maze Tests scores. To adjust for the confounding effect, covariates including age range, geographic setting (urban vs rural), school level among adolescent girls (primary vs secondary level), availability of potable water in schools, number of teachers at each school, parental status, and availability of school feeding program were used. The mean number of 24 for teachers across the groups (range: 5–32 teachers per school) was used as a cut-off to represent a proxy for large and small schools. Since we did not have the total number of pupils for each school, we could not calculate the teacher-student ratio.

For descriptive statistics, continuous variables were summarized using mean and standard deviation while categorical variables were summarized using frequency and percentages. In descriptive statistics, continuous variables were summarized using mean and standard deviation, while categorical variables were summarized using frequency and percentages. When an underlying health condition is rare (<10%), odd ratios precisely approximate relative risk since the estimates’ differences are negligible. However, since the prevalence of those with declined cognitive ability was more than 10% in our study, we anticipated inflation of the risk (and its confidence bound) using logistic regression [29–31]. Adjusted Poisson regression relies on robust standard errors to prevent inflation and unacceptable shrinkage of the confidence bound, thus providing a credible effect estimate. A robust standard error estimation technique was used in the modified Poisson regression model to correct the overestimation of the coefficient of association between exposures and outcome variables. The reference group was chosen based on the group with possible lowest risk of the outcome variable. All independent variables with p-value <0.05 in the bivariate analysis were entered in a multivariable model to adjust for the confounding effect (*see S3A and S3B Table for the unadjusted association*). The strength of association was expressed using prevalence ratio (PR) with their 95% confidence intervals. Independent variables with p-value <0.05 in multivariable analysis were considered as statistically significantly associated with the outcomes of interest.

### 2.5 Ethical clearance

The study protocol was reviewed and approved by the Catholic University of Health and Allied Sciences and Bugando Medical Centre’s Joint Ethics and Research Review Committee (*CREC/392/2019*). In addition, permission to conduct the study in the Simiyu Region was obtained from the regional administrative secretary as per Tanzanian Administrative protocols. Written consent for those aged ≥18 years and assent for those aged <18 years was prepared in Swahili. In addition, individual caregivers and/or guardians completed a written informed consent on behalf of their adolescent women who were aged <18 years. This consent and assent forms explained the purpose, risks, significance, and the right to either participate or withdraw from the study. Individual adolescent girls were given a unique identification code by their respective school matron, and no names were used to keep track of the participants without using identifiable information. This was done to reduce the risk of connecting the participant information to the individual participant.

## 3. Results

### 3.1 Socio-demographic characteristics of study participants

The characteristics of the participants are shown in Table 1. Most of the adolescent girls (IFA supplemented: n = 238, 60.1%, un-supplemented: n = 201, 53.74) who participated in this evaluation study were aged 11–19 years. Their mean age was 14.97 (±1.65) years. Irrespective of their IFA supplementation status, the majority (56.8%) of the participants were in secondary school and were living with both parents (78.7%). There were no significant differences in terms of age group (*χ*^2^ = 3.37, p = 0.180), and parental status (*χ*^2^ = 4.44, p = 0.110), for adolescent girls in the supplemented groups and un-supplemented group. However, adolescent girls school level (*χ*^2^ = 7.85, p = 0.010), parental economic activities (*χ*^2^ = 1.55, p = 0.001), residences (*χ*^2^ = 3.77, p = <0.001), availability of water (*χ*^2^ = 1.67, p<0.001), and feeding program (*χ*^2^ = 3.94, p = 0.010), at school, as well as number of teachers (*χ*^2^ = 4.69, p = 0.040), were statistically different with respect to IFA supplementation status (Table 1).

**Table 1:** Socio-demographic characteristics of the study participants by folic acid supplementation status (N = 770).

Variables			Supplementation Status				
	Overall (n = 770)		Supplemented (n = 396)		Not Supplemented (n = 374)		
	n	%	N	%	n	%	Test
*Age of Adolescent girls (years)*							
<12	21	2.73	11	2.78	10	2.67	χ^2^ = 3.37
12–15	439	57.01	238	60.10	201	53.74	
16–19	310	40.26	147	37.12	163	43.59	
Mean ±SD	15 ± 1.65						
*School level for adolescent girls*							
Primary	333	43.25	152	38.38	181	48.40	χ^2^ = 7.85*
Secondary	437	56.75	244	61.62	193	51.60	
*Parental main economic activities*							
Peasant	639	82.99	312	78.79	327	87.43	χ^2^ = 1.55*
Business	83	10.78	50	12.62	33	8.82	
Civil servant	48	6.23	34	8.59	14	3.75	
*Parental status*							
Both parents	606	78.70	315	79.55	291	77.81	χ^2^ = 4.44
Single parent	90	11.69	38	9.60	52	13.90	
Relative/orphans	74	9.61	43	10.85	31	8.29	
*Residence*							
Rural	496	64.42	154	38.89	342	91.44	χ^2^ = 3.77**
Urban	274	35.58	242	61.11	32	8.56	
*Availability of water at school*							
Available	545	70.78	321	81.06	224	59.89	χ^2^ = 1.67**
Not available	225	29.22	75	18.94	150	40.11	
*Availability of Feeding program at school*							
Available	83	10.78	18	4.55	65	17.38	χ^2^ = 3.94*
Not available	687	89.22	378	95.45	309	82.62	
*Number of teachers at school*							
<24	379	49.22	66	16.67	313	83.69	χ^2^ = 4.69*
≥24	391	50.78	330	83.33	61	16.31	

SD- standard deviation, χ^2^- Chi -squared test

### 3.2 Cognitive performance among adolescent girls by iron and folic acid supplementation status

Table 2 summarizes cognitive performance among adolescent girls in schools with and without IFA. There was a statistically significant difference in Span-backward (*χ*^2^ = 45.61%, p<0.001) and Span-forward (*χ*^2^ = 46.34%, p<0.001) cognitive performance where adolescent girls from schools with IFA supplementation performed much better as compared to schools without IFA supplementation. There was also a difference in Maze Test performance between adolescent girls from schools with IFA versus non-IFA supplemented schools even though this difference was not statistically significant (*χ*^2^ = 0.43%; p = 0.89). Overall, the girls performed better on the Span-backward and Span-forward than the Maze Test. There was a statistically significant difference in GCCP between adolescent girls from schools with IFA versus non-IFA supplemented schools those (*χ*^2^ = 32.52% p<0.001).

**Table 2:** Cognitive performance among adolescent girls by iron and folic acid supplementation status (N = 770).

Variables	Supplementation status						
	Overall (n = 770)		Supplemented (n = 396)		Not supplemented (n = 374)		
	n	%	n	%	n	%	Test
*Span—backward Test*							
Yes	265	34.42	224	56.57	41	10.96	*χ*^2^ = 45.61%**
No	505	65.58	172	43.43	333	89.04	
*Span—forward Test*							
Yes	307	39.87	247	62.37	60	16.04	*χ*^2^ = 46.34%**
No	463	60.13	149	37.63	314	83.96	
*Maze Test*							
Yes	645	83.77	331	83.59	314	83.96	*χ*^2^ = 0.43%
No	125	16.23	65	16.41	60	16.04	
*Composite cognitive ability*							
Yes	172	22.34	151	38.13	21	5.61	*χ*^2^ = 32.52%**
No	598	77.66	245	61.87	353	94.39	

**p-value <0.001

### 3.3 Cognitive abilities and IFA supplementation

#### Span-backward Test (Table 3)

In the adjusted model, school level for adolescent girls, parental status, residence, and presence of water at school premises were significantly associated with WIFAS. Participants with WIFAS had a 43% higher Span-backward ability (aPR = 1.43, 95% CI = 1.06–1.62) for those in secondary school as compared to those in primary school. Participants living with both parents had a 22% higher Span-backward ability (aPR = 1.22, 95% CI = 1.07–1.68) provided they received WIFAS as compared to those living with their relatives/orphans. Regarding residences, adolescent girls with WIFAS had a 29% higher Span-backward ability (aPR = 1.29, 95% CI = 1.06–1.41) in urban areas as compared to rural areas. In addition, adolescent girls with WIFAS, had a 34% higher Span-backward ability (aPR = 1.34, 95% CI = 1.03–1.89) if there was presence of water source at school as compared to those in schools without water sources.

**Table 3:** Adjusted association between Span-backward and Span-forward score and covariates by Iron and Folic Acid (IFA) supplementation status.

Variables	*Span—backward test*			*Span—forward test*		
	Overall	Supplemented	Not Supplemented	Overall	Supplemented	Not Supplemented
	aPR(95%CI)	aPR(95%CI)	aPR(95%CI)	aPR(95%CI)	aPR(95%CI)	aPR(95%CI)
*Age of adolescent girls (years))*						
<12	Ref	Ref	Ref	Ref	Ref	Ref
12–15	**1.18(1.02,1.36)** ^*^	2.15(0.84,5.50)	0.44(0.06,3.21)	1.05(0.91,1.22)	1.05(0.57,1.93)	0.92(0.13,6.65)
16–19	**1.20(1.01,1.37)** ^*^	2.11(0.80,5.5,4)	0.61(0.07,4.89)	1.02(0.87,1.18)	0.91(0.48,1.72)	0.98(0.13,7.41)
*School level for adolescent girls*						
Primary	Ref	Ref	Ref	Ref	Ref	Ref
Secondary	0.96(0.91,1.01)	**1.43(1.06,1.62)** ^*^	0.45(0.04,4.67)	1.03(0.98,1.08)	**1.26(1.04,1.53)** ^*^	0.67(0.28,1.60)
*Parental main economic activity*						
Peasant	Ref	Ref	Ref	Ref	Ref	Ref
Business	1.12(0.90,1.67)	1.13(0.04,1.98)	0.72(0.32,1.64)	1.21(0.43,1.78)	0.96(0.76,1.22)	0.48(0.14,2.94)
Civil servant	1.34(0.87,1.89)	1.21(0.05,1.83)	0.67(0.62,1.15)	1.06(0.95,1.89)	1.19(0.05, 1.64)	0.43(0.14,5.17)
*Parental status*						
Both parents	1.17(0.23,1.98)	**1.22(1.07,1.68)** ^*^	0.68(0.26,1.80)	**1.13(1.06,1.51)** ^*^	1.11(0.06,1.25)	1.09(0.59,2.02)
Single parent	1.02(0.78,1.29)	1.09(0.31,1.81)	0.82(0.48,2.16)	1.07(0.73,1.37)	1.08(0.05,1.18)	0.46(0.19,1.29)
Living to relative/orphans	Ref	Ref	Ref	Ref	Ref	Ref
*Residence*						
Rural	Ref	Ref	Ref	Ref	Ref	Ref
Urban	**1.25(1.09, 1.71)** ^*^	**1.29(1.06,1.41)** ^*^	0.73(0.68,3.65)	1.15(0.81,1.79)	1.14(0.03,1.17)	0.36(0.22,0.60)
*Availability of water at school*						
Available	0.95(0.89,1.01)	**1.34(1.03,1.89)** ^*^	0.37(0.05,2.55)	0.93(0.88,1.99)	1.02(0.77,1.35)	0.69(0.31,1.52)
Not available	Ref	Ref	Ref	Ref	Ref	Ref
*Availability of feeding program at school*						
Available	**1.14(1.06,1.24)** ^*^	1.20(0.04,1.28)	0.78(0.54,3.85)	**1.25(1.07,1.73)** ^*^	**1.38(1.03,1.63)** ^*^	0.39(0.04,3.02)
Not available	Ref	Ref	Ref	Ref	Ref	Ref
*Number of teachers at school*						
<24	Ref	Ref	Ref	Ref	Ref	Ref
≥24	**1.09(1.03,1.16)** ^*^	1.35(0.21,2.76)	0.36(0.13,1.01)	1.01(0.95,1.07)	**1.28(1.02,1.74)** ^**^	0.17(0.05,0.52)

aPR-adjusted prevalence ratio, CI-confidence interval, the models were adjusted for all the covariates *p<0.05

**p<0.001

#### Span-forward test (Table 3)

In the adjusted model, school level for adolescent girls, feeding program at school and number of teachers at school were significantly associated with Span-forward ability among adolescent girls with WIFAS as compared to those who were not supplemented. Adolescent girls in secondary school level of education had a 26% higher Span-forward ability (aPR = 1.26, 95% CI = 1.04–1.53) as compared to those in primary school level of education if receiving WIFAS. Adolescent girls with WIFAS scored 38% higher on the Span-forward test (aPR = 1.38, 95% CI = 1.03–1.63) as compared to those with no feeding program at their school. Adolescent girls had 28% higher Span-forward ability (aPR = 1.28, 95% CI = 1.02–1.74) at schools with teachers above the average of 24 teachers as compared to those with less than the average number of teachers.

#### Maze Test (Table 4)

For maze ability, adolescent girls who were in secondary school level of education had a 12% higher cognitive ability (aPR = 1.12, 95% CI = 1.01–1.25) compared to those in primary school for the schools with IFA supplementation. Adolescent girls with WIFAS had an 18% higher cognitive ability for the Maze Test (aPR = 1.18, 95% CI = 1.04–1.55) when in urban areas compared to those residing in rural areas. Adolescent girls supplemented with IFA had a 15% higher Maze Test score (aPR = 1.15, 95% CI = 1.07–1.26) when there was a feeding program at school.

**Table 4:** Adjusted association between maze ability score and Global Composite Cognitive Performance (GCCP) score and covariates by Iron and Folic Acid (IFA) supplementation.

Variables	Maze ability			Composite cognitive ability			
	Overall	Supplemented	Not Supplemented	Overall		Supplemented	Not Supplemented
	aPR(95%CI)	aPR(95%CI)	aPR(95%CI)	aPR(95%CI)	aPR(95%CI)		aPR(95%CI)
*Age of Adolescent girls (years)*							
<12	Ref	Ref	Ref	Ref	Ref		Ref
12–15	1.03(0.92,1.16)	1.33(0.73,1.39)	0.33(0.08, 2.25)	2.4(0.67,8.42)	1.02(0.91,1.14)		0.78(0.41,1.15)
16–19	1.01(0.89,1.14)	1.24(0.70,1.30)	0.64(0.03,2.11)	2.10(0.57,7.53)	1.11(0.81,1.52)		0.16(0.01,1.56)
*Education level*							
Primary	Ref	Ref	Ref	Ref	Ref		Ref
Secondary	**1.07(1.01,1.09)** ^*^	1.12(1.01,1.25)^*^	0.99(0.81,1.20)	1.20(0.89,1.62)	1.32(0.07,1.80)		0.21(0.18,2.77)
*Parental main economic activity of the parents*							
Peasant	Ref	Ref	Ref	Ref	Ref		Ref
Business	0.67(0.32,0.97)^*^	1.07(0.95, 1.21)	0.99(0.85,1.16)	1.04(0.51,1.89)	**1.23(1.11,1.76)** ^*^		0.88(0.23,9.25)
Civil servant	1.03(0.91,1.84)	1.12(0.98,1.26)	0.86(0.50,1.25)	**1.21(1.12,1.67)** ^**^	**1.17(1.03,1.41)** ^*^		0.58(0.14,1.08)
*Parental status*							
Both parents	1.23(0.92,1.56)	1.08(0.93,1.25)	0.82(0.29,1.15)	**1.15(1.09,1.63)** ^**^	1.62(0.32,1.79)		0.32(0.04,2.44)
Single parent	0.94(0.82,1.19)	1.03(0.90,1.18)	0.35(0.10,1.22)	**1.12(1.04,1.82)** ^*^	1.03(0.22, 1.85)		0.63(0.17,2.31)
Relative/orphans	Ref	Ref	Ref	Ref	Ref		Ref
*Residence*							
Rural	Ref	Ref	Ref	Ref	Ref		Ref
Urban	1.06(1.02,1.71)	**1.18(1.04,1.55)** ^*^	0.98(0.81,1.18)	0.98(0.45,1.98)	1.29(0.16,1.76)		0.28(0.07,1.97)
*Availability of water at school*							
Available	0.94(0.91,2.98)	1.11(0.69,1.95)	1.01(0.83,1.21)	**1.57(1.38,1.86)** ^*^	**1.27(1.03,1.75)** ^*^		0.79(0.20,3.16)
Not available	Ref	Ref	Ref	Ref	Ref		Ref
*Availability of Feeding program at school*							
Available	**1.08(1.01,1.09)** ^*^	**1.15(1.07,1.26)** ^*^	0.90(0.66,1.26)	**1.34(1.12,3.56)** ^*^	1.07(0.61,1.89)		0.75(0.56,4.04)
Not available	Ref	Ref	Ref	Ref	Ref		Ref
*Number of teachers at school*							
<24	Ref	Ref	Ref	Ref	Ref		Ref
≥24	0.98(0.93,1.02)	1.15(0.02,1.41)	0.88(0.74, 1.05)	**1.56(1.02,2.38)** ^*^	2.01(1.12,3.62)^*^		0.27(0.07,1.08)

aPR-adjusted prevalence ratio

*-p-value<0.05

** p-value <0.001

#### Global Composite Cognitive Performance (GCCP)(Table 4)

After adjusting for other variables, parental status, availability of water at school, and number of teachers at school were significantly associated with composite cognitive ability for participants who received WIFAS as compared to those who were not supplemented. Adolescent girls with parents who worked as civil servants had a 17% higher GCCP ability (aPR = 1.17, 95% CI = 1.03–1.41) when compared to those with parents working in farming/peasant. Adolescent girls with parents who were involved with business activities had a 23% higher GCCP (aPR = 1.23, 95% CI = 1.11–1.76) as compared to those with parents working in farming/peasant. Adolescent girls supplemented with IFA had a 27% higher GCCP ability (aPR = 1.27, 95% CI = 1.03–1.75) when there was presence of water source at their school. Furthermore, participants with more than 24 teachers (using the mean number of teachers across groups as the cut-off) in their schools had scores two times higher for GCCP ability score (aPR = 2.01, 95% CI = 1.12–3.62) as compared to their counterpart with less than 24 teachers at school.

## 4. Discussion

The economic, social, and psychological cost of cognitive decline is enormous and multidimensional. Research shows that iron deficiency (ID) related anemia and folic acid deficiency (FAD) are key determinants of nutrition-associated cognitive decline, especially among children and adolescents [32, 33]. The largest proportion of the 300 million global anemic cases among children are from Africa [34, 35], representing a significant health burden since many of these children may have resulting cognitive impairment. In our current study, we found support for the linkage between cognitive ability and IFA supplementation among adolescent girls aged 10–19 years in Itilima and Meatu Districts in Simiyu Region. Specifically, we found higher attention, short memory retention, and working memory among adolescent girls who were on IFA supplementation. Additionally, we observed higher levels of thinking and acting quickly for adolescent girls supplemented with IFA. These findings cannot overemphasis the need to advance nutrition-related public health interventions in Tanzania for IFA.

Even though the mechanism linking IFA with anemia and cognitive decline among adolescent girls is not fully understood, some studies suggest that inadequate cerebral oxygen delivery and oxygenation to the brain may offer a plausible answer [5]. For example, one study found a significant association between anemia and reduced cognitive ability [36]. Low concentrations of hemoglobin can contribute to chronic brain hypoxia and reduced aerobic capacity in the brain, thus causing severe brain injury and hence cognitive decline [36]. Additionally, since the brain has erythropoietin receptors that are involved in neuroprotection, hypoxia may cause decreased erythropoietin levels, which could raise the risk of neuronal degeneration in several cognitive pathways. This leads to impaired neurosynthesis and hence a decline in brain development and volume resulting in poor mental functioning [5, 6]. Since iron is crucial for brain oxygen transport, electron transfer, neurotransmitter synthesis, and myelin production [4], it makes logical sense that those with IFA supplementation had higher cognitive abilities. Another mechanism that is mainly linked to folic acid deficiency and cognitive decline may be the role of folate in DNA synthesis. For example, folate has been found to regulate nucleotide and DNA synthesis and histone methylation and therefore, a lack of folic acid may lead to changes in DNA synthesis and thus neurological disorders [37]. These changes are often linked to altered gene expression and might be linked to failed control of specific regulatory networks in the brain. This might explain the reason why folic acid deficiency has been associated with various diseases, including cancer and cognitive impairment [37].

Our current findings could not explain the etiology of lower cognitive functioning by the pathophysiologic mechanisms involving iron and folic roles. Besides IFA supplementation, age, availability of potable water at school, the presence of school feeding programs, numbers of teachers, living with a parent or family members, and level of school (secondary vs primary), all predicted how well an adolescent girl performed on various dimensions of the cognitive tests. This indicates that the level of cognitive ability is a function of a complex constellation of multiple factors that span nutrition, behavior, culture, sociodemographic, and genetic factors [38].

Cognitive abilities may improve with age because of concordance maturation of the brain toward reaching an adult-sized brain [39]. Further, higher cognitive abilities among adolescents in schools with higher-than-average numbers of teachers could be attributed to longer encounters with their teachers during curricular and extra-curricular activities or possibly more resources if a larger school. Access to teachers at school provided adequate time for students to spend with their teachers [25]. Evidence elsewhere suggests that, found that pupils who had more teachers at school had better learning outcomes and a better teaching environment as compared to pupils with fewer [40]. In these studies, it was proposed that increased contact with teachers helped students to ask a wide range of questions and solved problems related to other extracurricular activities, which in turn resulted in higher cognitive abilities.

Whether a school is in an urban or rural setting influences adolescent girls’ cognitive performance, as those from urban schools were more likely to out-perform those from rural areas on various tests. Children from urban schools may have increased access to school feeding programs, more social amenities, and a higher number of teachers with more training [41, 42]. This points to the need to develop policies that minimize inequalities in the distribution and redistribution of teachers across Tanzania, ensuring that schools are staffed with enough highly trained teachers to serve the student population.

As suggested by our adjusted model, having access to potable water and school feeding programs resulted in higher cognitive performance among our participants. Some studies have shown that dehydration can affect biological domains and can reduce short-term memory and perception [43, 44]. Further, school feeding programs across many countries in Africa, including Ghana [45] and Kenya [46] have proven helpful in school enrollment and retention. Having clean water and free food in schools may not only increase enrollment and attendance but will also up turn students’ contact hours with their teachers. Increasing contact hours may help students engage in various problem-solving-oriented activities which in turn improves their cognitive abilities.

The home environment is also important for cognitive development, including availability of food and clean water. Participants living with one or both parents had higher cognitive abilities as compared to orphans or those living with their relatives. Dual parent households often mean that food and healthcare needs are more easily met. In addition, orphans may be under emotional stress and might be deprived of basic needs like food, hence poor physical and mental development [47]. Orphans are overwhelmed with family responsibilities and school duties compared to non-orphan adolescents [47].

Taking all factors together, we encourage multi-level public health interventions that improve nutritional access and equity-based education policy in terms of human and food resources supply. Further, we suggest community education programs on the parental role in child development. Importantly, in low-and middle-income countries, not all adolescents are enrolled in schools [47] and so would not benefit from school based WIFAS. As such, community-based programs for IFA supplementation would reach many more children and adolescents. For example, encouraging a supplement when making porridge, a very common meal for children and adolescents particularly in villages, could improve IFA access and uptake.

### 4.1 Strengths and limitations of the study

To the best of our knowledge, this is the first study in Tanzania to investigate the role of iron and folic use on cognitive abilities among adolescent girls. Our study provides insights to guide the course of targetable public health intervention at the individual and population levels. However, our study was not free from limitations. First, the study did not provide a mechanistic link between anemia and reduced cognitive ability, particularly with regard to IFA. Studies on the association between IFA and declined cognitive ability are minimal [36]. Secondly, individual haemoglobin status was not assessed to measure its association with cognitive abilities, nor was baseline cognitive functioning assessed to evaluate whether there was a change. Haemoglobin is proxy indicator of anemia which has been associated with cognitive impairment [36]. Even though the prevalence of iron deficiency anemia among adolescent girls in northwestern Tanzania is not well established, the prevalence of iron deficiency anemia among women of reproductive age is around 53% providing some insights. Follow up studies need to consider establishing baseline values for haemoglobin anemia along with cognitive assessments before and after an intervention. Thirdly, even though the present study did not compare the significance between the two tests, previous studies used Digit Span Test and Maze Test to assess cognitive performance among adolescent girls elsewhere reported the two tests being valid [27]. Although socioeconomic status relates to nutritional status [24, 38, 48], with a potential impact on cognitive abilities, we could not adequately control this factor in our adjusted model as this was a school-based study. Individual family socio-economic status affects the number and quality of meals a family can afford and may affect their micronutrient deficiency, such as iron. Follow up studies should consider integrating family assessment. Even though our findings are supported by previous evidence from the literature, they cannot be generalized to all adolescent women in other settings. Further, there are numerous causes of iron and folic acid deficiency, including nutritional, altitude, infections such as malaria and intestinal parasitic infection, chronic infection, and settings, which all should be considered when considering the risk factors for lower cognitive functioning [47].

## 5. Conclusion

The economic, social, and psychological cost of reduced cognitive function impairment is enormous and multidimensional. The present study provides compelling evidence that WIFAS is positively associated with higher cognitive ability among adolescent girls. Changing nutritional status of adolescent girls due to social, cultural, and economic reasons is extremely challenging and so intermittent, but consistent, IFA supplementation represents a relatively simpler and cost-effective solution that is crucial to ensure adolescent girls achieve their highest cognitive potential. Further, we recommend appropriate public health education on dietary diversification to improve the intake of iron and folate-rich diets that can alleviate anemia and positively affect cognitive abilities for adolescent girls. This calls for investment in adolescent health and nutrition through community-wide programs.

## Supporting information

S1 Checklist STROBE checklist. (DOCX)

S1 Table Selection of schools and adolescents women aged 10–19 for participation in the study. (DOCX)

S2 Table Digit Span Test table. (DOCX)

S3 Table a: Unadjusted association between Span-backward and Span-forward and the predictor variables among study participants by iron and folic acid supplementation status.b: Unadjusted association between maze ability and composite cognitive ability and the exposure variables by iron and folic acid supplementation status.
(DOCX)

S1 Dataset (CSV)
