# Supplementary material for: Influence of intermittent iron and folic acid supplementation on cognitive abilities among adolescent girls in northwestern Tanzania
Source: PLOS Glob Public Health. 2023 Oct 18;3(10):e0002079. doi: 10.1371/journal.pgph.0002079 (PMC10584093; doi:10.1371/journal.pgph.0002079)
Supplement: S1 Table — (DOCX) [file pgph.0002079.s002.docx]

S1 Table: Selection of schools and Adolescents women aged 10-19 for participation in the study

| Domain | Strata | | | Interview by Strata | | | Number of Schools by Strata | | | Interview per School |
| --- | --- | --- | --- | --- | --- | --- | --- | --- | --- | --- |
|  | Secondary | Primary | Total | Secondary | Primary | Total | Secondary | Primary | Total |  |
| Itilima | 30 | 119 | 149 | 247 | 153 | 400 | 8 | 7 | 15 | 27 |
| Meatu | 23 | 112 | 135 | 221 | 179 | 400 | 8 | 7 | 15 | 27 |
| Total | 53 | 231 | 254 | 468 | 332 | 800 | 16 | 14 | 30 | 54 |

**Note:** *Even though the expected minimum number of adolescent girls per school was around 27 per each of the selected schools, the actual number was adjusted based on the exact number of adolescent girls present during the school visit. In Itilima District were as follows for primary schools; Lagangabilili, Ikungulipu, Migato, Mwamtani, Mwaswale, Ndoleleji, and Nkoma A primary schools with 20, 24, 21, 22, 25, 22, and 19 respectively. For secondary schools were as follows, Bunamhala (33), Ikungulipu (28) Itilima (31), Lagangabilili (31), Kanadi (30), Mhunze (31)), Mwaswale (32) and Shishani (31).*

*In Meatu District, the number of adolescent girls in each of the selected schools was indicated in brackets per school; Primary schools - Mshikamano (26), Mwanhuzi (25), Itinje (26), Kisesa (25), Mwabuzo (25), Mwabuma (25) and Mwamanongu (27). For a secondary school in Meatu selected adolescents girls were as follows; Mwanyahina (27), Mwangudo (21), Meatu (29), Bukundi (30), Paji (28), Mwamishali (29), Mwandoya (28), Ngoboko (29).*
