# Supplementary material for: Influence of intermittent iron and folic acid supplementation on cognitive abilities among adolescent girls in northwestern Tanzania
Source: PLOS Glob Public Health. 2023 Oct 18;3(10):e0002079. doi: 10.1371/journal.pgph.0002079 (PMC10584093; doi:10.1371/journal.pgph.0002079)
Supplement: S2 Table — (DOCX) [file pgph.0002079.s003.docx]

S2 Table: Digit span test table
