# Supplementary material for: Influence of intermittent iron and folic acid supplementation on cognitive abilities among adolescent girls in northwestern Tanzania
Source: PLOS Glob Public Health. 2023 Oct 18;3(10):e0002079. doi: 10.1371/journal.pgph.0002079 (PMC10584093; doi:10.1371/journal.pgph.0002079)
Supplement: S3 Table — a: Unadjusted association between Span-backward and Span-forward and the predictor variables among study participants by iron and folic acid supplementation status.b: Unadjusted association between maze ability and composite cognitive ability and the exposure variables by iron and folic acid supplementation status. (DOCX) [file pgph.0002079.s004.docx]

S3 Table a: Unadjusted association between Span-backward and Span-forward and the predictor variables among study participants by iron and folic acid supplementation status.

| Variables | **Span-backward Span-forward** | | | | | | |
| --- | --- | --- | --- | --- | --- | --- | --- |
|  | Not  Overall Supplemented Supplemented | | | | Not  Overall Supplemented Supplemented | | |
|  | cPR(95%CI) | cPR(95%CI) | | cPR(95%CI) | cPR(95%CI) | cPR(95%CI) | cPR(95%CI) |
| *Age of Adolescent girls (years)* | |  | |  |  |  |  |
| <12 | Ref | Ref | | Ref | Ref | Ref | Ref |
| 12-15 | 1.16(0.99,1.34) | 1.26(0.02,1.56) | | 0.99(0.84,1.19) | 1.07(0.92,1.25) | 1.18(0.06,2.04) | 0.97(0.27,1.80) |
| 16-19 | 1.10(0.95,1.27) | 1.19(0.96,1.48) | | 0.82(0.16,1.22) | 1.02(0.87,1.19) | 1.09(0.03,1.91) | 0.71(0.21,9.44) |
| *School Level of adolescent girls* | |  | |  |  |  |  |
| Primary | Ref | Ref | | Ref | Ref | Ref | Ref |
| Secondary | 0.99(0.95,1.05) | 1.05(1.01,1.22)^*^ | | 0.98(0.92,1.04) | 1.03(0.98,1.08) | 1.13(0.05,1.33) | 0.63(0.39,1.01) |
| *Main economic activity of the parents* | |  | |  |  |  |  |
| Peasant | Ref | Ref | | Ref | Ref | Ref | Ref |
| Business | 1.01(0.91,1.12) | 1.16(0.03, 1.31) | | 0.90(0.75,1.08) | 0.79(0.33,1.67) | 1.01(0.81,1.26) | 0.71(0.56,2.62) |
| Civil servant | 1.02(0.90,1.16) | 1.06(0.91, 1.23) | | 0.99(0.81,1.23) | 0.95(0.81,1.45) | 1.18(0.56,1.61) | 0.39(0.13,1.04) |
| *Parent status* |  |  | |  |  |  |  |
| Both parents | 1.19(1.03,1.93)^*^ | 1.17(1.06,1.98)^*^ | | 0.97(0.90,1.04) | 0.98(0.67,1.86) | 1.26(1.04,1.75)^*^ | 0.14(0.03,2.21) |
| Single parent | 1.33(0.11,1.80) ^*^ | 1.09(1.01,1.86)^*^ | | 1.05(0.93,1.17) | 0.56(0.22,1.49) | 1.09(0.02,1.61) | 0.19(0.11,1.82) |
| Relative/orphans | Ref | Ref | | Ref | Ref | Ref | Ref |
| *Residence* |  |  | |  |  |  |  |
| Rural | Ref | Ref | | Ref | Ref | Ref | Ref |
| Urban | 1.17(.76,1.84)^**^ | 1.12(1.08,1.15)^*^ | | 0.92(0.86,0.98) | 0.92(0.78,1.86) | 1.46(1.02,1.91)^*^ | 0.58(0.12,1.65) |
| *Availability of water at school* | |  | |  |  |  |  |
| Available | 1.17(1.01,1.53) ^*^ | 1.19(1.02,1.28)^*^ | | 0.95(0.90,1.01) | 1.05(0.99,1.11) | 1.05(0.85,1.28) | 0.51(0.32,0.82) |
| Not available | Ref | Ref | | Ref | Ref | Ref | Ref |
| *Availability of feeding program at school* | | | |  |  |  |  |
| Available | 0.97(0.89,1.06) | 1.25(0.05, 1.76) | | 0.99(0.85,1.15) | 0.91(0.84,1.99) | 1.17(1.06,1.50)^*^ | 0.89(0.66,2.11) |
| Not available | Ref | Ref | | Ref | Ref | Ref | Ref |
| *Number of teachers at school* | | |  | |  |  |  |
| <24 | Ref | Ref | Ref | | Ref | Ref | Ref |
| ≥24 | 1.32(1.26,1.38)^*^ | 1.23(1.12,1.34)^*^ | 0.92(0.87,0.97) | | 1.26(1.20,1.32)^**^ | 1.39(1.09,1.82)^*^ | 0.17(0.04,0.71) |

cPR-crude prevalence ratio, *-p-value<0.05, ** p-value <0.001

S3 Table b: Unadjusted association between maze ability and composite cognitive ability and the exposure variables by iron and folic acid supplementation status

| Variables | **Maze ability Composite cognitive ability** | | | | | |
| --- | --- | --- | --- | --- | --- | --- |
|  | Not  Overall Supplemented Supplemented | | | Not  Overall Supplemented Supplemented | | |
|  | cPR(95%CI) | cPR(95%CI) | cPR(95%CI) | cPR(95%CI) | cPR(95%CI) | cPR(95%CI) |
| *Age of Adolescent girls (years)* | |  |  |  |  |  |
| <12 | Ref | Ref | Ref | Ref | Ref | Ref |
| 12-15 | 1.07(0.96,1.20) | 1.01(0.75,1.34) | 0.43(0.05,2.37) | 2.60(0.68, 9.76) | 2.19(0.62,7.78) | 0.56(0.32,2.56) |
| 16-19 | 1.08(0.95,1.21) | 1.045(0.78,1.39) | 0.39(0.03,2.32) | 2.10(0.55,8.00) | 2.02(0.56,7.22) | 0.67(0.56,1.24) |
| *School level of adolescent girls* | |  |  |  |  |  |
| Primary | Ref | Ref | Ref | Ref | Ref | Ref |
| Secondary | 1.43(1.04,1.86)^*^ | 1.39(1.01, 1.72)^*^ | 0.74(0.25,1.14) | 1.29(0.98,1.69) | 1.26(0.95,1.65) | 0.49(0.19,1.14) |
| *Main parental economic activity* | |  |  |  |  |  |
| Peasant | Ref | Ref | Ref | Ref | Ref | Ref |
| Business | 0.90(0.65,0.97)^*^ | 1.07(0.95,1.20) | 0.82(0.67,1.18) | 0.94(0.43,1.69) | 0.79(0.52, 1.21) | 0.64(0.43,1.51) |
| Civil servant | 1.34(0.02,1.56) | 1.11(0.99, 1.25) | 0.11(0.05,1.29) | 1.12(1.05,2.34) | 1.05(0.37,1.17) | 0.81(0.78,1.34) |
| *Parental status* |  |  |  |  |  |  |
| Both parents | 1.08(0.43,1.76) | 1.05(0.92,1.19) | 0.41(0.19,1.15) | 1.39(1.17,1.78)^*^ | 1.19(1.02,1.21) ^*^ | 0.64(0.03,7.51) |
| Single parent | 1.02(0.08,1.65) | 1.04(0.91, 1.18) | 0.74(0.40,1.21) | 1.18(1.06,1.88)^*^ | 1.15(0.07,1.17) | 0.11(0.08,1.34) |
| Relative/orphans | Ref | Ref | Ref | Ref | Ref | Ref |
| Residence |  |  |  |  |  |  |
| Rural | Ref | Ref | Ref | Ref | Ref | Ref |
| Urban | 1.08(0.67,1.43) | 1.12(0.02,1.79) | 0.99(0.85,1.16) | 1.18(1.01,1.59)^**^ | 1.23(1.05,1.80)^*^ | 0.77(0.59,1.02) |
| *Availability of water at school* | |  |  |  |  |  |
| Available | 0.97(0.95,1.03) | 1.15(0.06,1.24) | 0.80(0.71,1.69) | 1.10(0.82,1.48) | 1.10(0.60,1.27) | 0.41(0.17,0.97) |
| Not available | Ref | Ref | Ref | Ref | Ref | Ref |
| *Availability of Feeding program at school* | | |  |  |  |  |
| Available | 1.24(1.02,1.68)^*^ | 1.47(1.04,1.56)^**^ | 1.01(0.30,1.21) | 1.36(0.83, 2.24) | 1.22(1.06,1.85)^**^ | 0.37(0.09,5.66) |
| Not available | Ref | Ref | Ref | Ref | Ref | Ref |
| *Number of teachers at school* | |  |  |  |  |  |
| <24 | Ref | Ref | Ref | Ref | Ref | Ref |
| ≥24 | 0.98(0.95, 1.01) | 1.28(1.03,1.63)^**^ | 0.86(0.73,1.00) | 2.24(1.74,2.89)^**^ | 2.50(1.46,4.43)^*^ | 0.26(0.03,1.88) |

cPR-crude prevalence ratio, *-p-value<0.05, ** p-value <0.001
